# The role and mechanism of CRL4 E3 ubiquitin ligase in cancer and its potential therapy implications

**DOI:** 10.18632/oncotarget.6052

**Published:** 2015-10-09

**Authors:** Youzhou Sang, Fan Yan, Xiubao Ren

**Affiliations:** ^1^ Department of Immunology, Tianjin Medical University Cancer Institute and Hospital, Tianjin, China; ^2^ Department of Biotherapy, Tianjin Medical University Cancer Institute and Hospital, Tianjin, China; ^3^ National Clinical Research Center of Cancer, Tianjin, China; ^4^ Key Laboratory of Cancer Immunology and Biotherapy, Tianjin, China

**Keywords:** CRL4, CUL4, ubiquitination, cancer

## Abstract

CRLs (Cullin-RING E3 ubiquitin ligases) are the largest E3 ligase family in eukaryotes, which ubiquitinate a wide range of substrates involved in cell cycle regulation, signal transduction, transcriptional regulation, DNA damage response, genomic integrity, tumor suppression and embryonic development. CRL4 E3 ubiquitin ligase, as one member of CRLs family, consists of a RING finger domain protein, cullin4 (CUL4) scaffold protein and DDB1–CUL4 associated substrate receptors. The CUL4 subfamily includes two members, CUL4A and CUL4B, which share extensively sequence identity and functional redundancy. Aberrant expression of CUL4 has been found in a majority of tumors. Given the significance of CUL4 in cancer, understanding its detailed aspects of pathogenesis of human malignancy would have significant value for the treatment of cancer. Here, the work provides an overview to address the role of CRL4 E3 ubiquitin ligase in cancer development and progression, and discuss the possible mechanisms of CRL4 ligase involving in many cellular processes associated with tumor. Finally, we discuss its potential value in cancer therapy.

## INTRODUCTION

Ubiquitin-proteasome pathway plays a vital role in the degradation of proteins, which is mediated by the successive enzymatic reactions involving in a ubiquitin-activating enzyme (E1), a ubiquitin-conjugating enzyme (E2), and a ubiquitin ligase (E3) [1]. In this process, the ubiquitin ligase determines the substrate specificity. Among the diverse E3 enzymes, the cullin-RING ubiquitin ligases (CRLs) are the largest E3 ligase family in eukaryotes [2]. The cullin as a scaffold protein binds with its C-terminal region to a RING-finger protein RBX1/ROC1, whereas its N-terminus associates with a cullin-specific adaptor protein to recruit a large family of substrate receptors, each of which can target diverse substrates [3]. CRLs activity can be regulated by a process termed neddylation that involves cullin-associated protein called Nedd8 [4]. Cullin neddylation is reverted by the COP9 signalosome (CSN) through a reaction known as deneddylation [5].

The human cullin family mainly comprises of eight intimately related proteins (CUL1, CUL2, CUL3, CUL4A, CUL4B, CUL5, CUL7 and CUL9). Compared to another cullins, the CUL4 subfamily includes two members, CUL4A and CUL4B, which share over 80% sequence identity and functional redundancy. Each cullin acts as a scaffold to interact with a specific adaptor and diverse substrate receptors. For instance, the CUL1-Rbx1 complex utilizes Skp1 as its adaptor to interact with a myriad of F-box proteins that act as substrate receptors [6]. However, CUL4 uses DNA damage binding protein 1 (DDB1) as adaptor and CUL4-associated factors (DCAFs) as substrate receptors to identify a large number of substrate proteins [2, 7, 8]. That the aberrant expression of CUL4 has been founded in many types of tumors suggests the close relationship between CUL4 and cancer [9, 10], and more and more researches recently have shown that CRL4 ligase is involved in some cellular processes associated with tumor, such as cell cycle regulation, signal transduction, transcriptional regulation, DNA damage response, genomic integrity, tumor suppression and so on [2]. This review provides insights for the role of CUL4 ligase in cancer development and progression, and summarizes current researches on the functional regulation of CUL4 in tumor-related changes, including cell cycle, DNA damage repair, histone methylation, signaling pathways, oncoproteins turnover. Additionally, potential therapeutic value of CUL4 in cancer is also discussed in the work.

## CRL4 E3 UBIQUITIN LIGASE IN CANCER

CUL4A and CUL4B share extensive homology and functional redundancy, however CUL4A has drawn much more attention as a result of a large body of evidence demonstrating its association with cancer. Recent years, accumulating studies have shown that CUL4A expresses abnormally in multiple cancers including breast cancer, squamous cell carcinoma, pleural mesothelioma [9-12], and one latest study found CUL4A overexpressed in non-small-cell lung cancer and stimulated the latter growth as well as tumorigenesis [13]. CUL4A overexpression contributes to tumor progression, metastasis, and poorer survival rate of cancer patients [14]. For example, Chen et al. showed that CUL4A amplified in lymph node-negative breast cancer, and these patients with CUL4 overexpression showed shorter overall and disease-free survival (OS and DFS) [15]. And a report found that CUL4A silencing in CUL4A-overexpressing breast cancer cells induced a reduction of cell proliferation and colony formation and decreased the tumor development [16]. These results suggest CUL4A may be a critical contributor to the progression and development of breast cancer. In addition, CUL4A knockout mice are resistant to UV-induced skin carcinogenesis [17], while overexpression of CUL4A in transgenic mouse lungs induces potential hyperplasia [18]. Compared to CUL4A, reports about the relationship between CUL4B and tumor are lack. Recent researches are mainly about that CUL4B is causally associated with human X-linked mental retardation [19]. Nevertheless, a recent work found that CUL4B overexpressed in colon cancer, and its overexpression closely related to tumor stage, histological differentiation, vascular invasion and distant metastasis. And patients with positive CUL4B expression had a lower OS and DFS rate than patients with negative CUL4B expression [20]. Meanwhile, recent studies showed that CUL4B upregulated in some other cancers such as cervix, lung, esophagus and breast cancers, which associated with tumor invasion and lymph node metastasis [21]. Furthermore, CUL4B overexpression promotes the development of spontaneous liver tumors at a high rate and enhances DEN-induced hepatocarcinogenesis in transgenic mice [22]. Consequently, based on the above facts, CRL4 E3 ligase plays a crucial role in cancer and represents an ideal target site for therapeutic intervention.

## THE POSSIBLE MECHANISM OF CRL4 E3 UBIQUITIN LIGASE INDUCING CANCER

**Figure 1:**
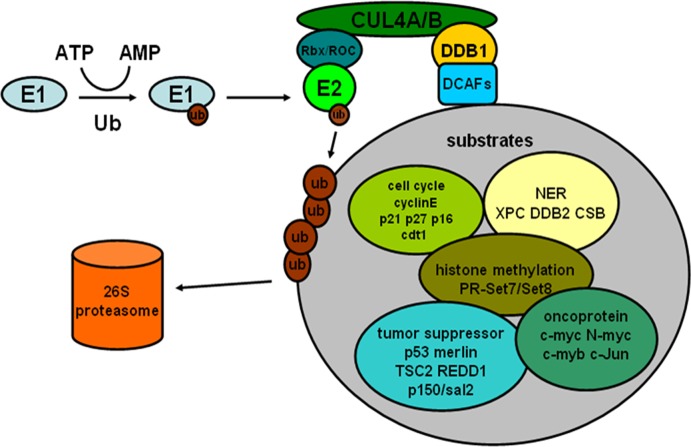
The process of ubiquitin-proteasome pathway and some substrates of CRL4 E3 ligases associated with cancer

It is becoming increasingly clear that through selective degradation of target proteins, CRL4 ligases participate in multiple physiological processes such as cell cycle, nucleotide excision repair and histone methylation. Furthermore, such physiological processes have great relevance with the development of human cancer. In addition, some substrates of CRL4 are involved in many signaling pathways related to cancinogenesis and include multiple oncoproteins. (Figure 1) The detailed mechanism of CRL4 ligase regulating the above processes will be discussed below.

## CRL4 AND CELL CYCLE

Based on a majority of studies, many researchers have shown that cancer can be regarded as a disease of the cell cycle. Deregulation of the cell cycle and genome instability are two common features of cancer cells [23], and various mechanisms exist to preserve the integrity of the genome and guard against cancer. In normal cells, cell cycle progression is strictly regulated by multiple mechanisms to ensure its orderly progress. Most researches consider that it is regulated mainly by the cell cycle proteins (Cyclins), cyclin-dependent kinases (CDKs) and cyclin-dependent kinase inhibitors (CDKIs). Consequently, regulation of the above proteins is crucial for cell cycle process, and more and more researches have found the expression level of these proteins was clearly associated with tumorigenesis and cancer progression. In recent years, accumulating evidence has identified that the turnovers of the above proteins are regulated by CRL4 ubiquitin ligase.

Cyclin E is a regulator of the G1 phase of the cell cycle and proper regulation of this cyclin is essential for S-phase transition and multiple processes safeguarding the accuracy of DNA replication [24]. Abnormal cyclin E expression has been found in several types of tumors. For example, altered expression of cyclin E occurs in the breast cancer and can serve as molecular marker which associates with the prognosis and therapy [25]. The overexpression of Cyclin E is also implicated in carcinomas at various sites along the gastrointestinal tract, especially the stomach and the colorectal region [26]. Consequently, the turnover level of cyclin E correlates with the pathogenesis of cancer. Many reports have suggested that cyclin E is tightly regulated by ubiquitin-mediated proteolysis system. Wild-type CUL4B-RING E3 ligase enhances the degradation of cyclin E in HEK 293 cells, while down-regulation of CUL4B results in a significant cyclin E accumulation compared with that of control cells [27, 28]. Similar results are observed in HeLa cells, and RNAi of CUL4B inhibits the proliferation of HeLa cells by prolonging S phase progression. These results suggest that the inhibiting effect of CUL4B on cell proliferation may be mediated by targeting cyclin E for degradation [27]. Interestingly, silencing of CUL4A also causes an increase in cyclin E levels [28]. Thus, aberrant expression of CUL4A/B contributes to unstable cyclin E level leading to unusual cell cycle, which may induce relevant cancer.

CDK inhibitor p21 is a critical effector in response to a variety of intracellular and extracellular stress signals. p21 can inhibit the kinase activity of Cdk2, thereby preventing progression into or through the S phase [29]. Since it was discovered, an abundance of researches have provided insights into the role of p21 as a regulator of tumor development and progression [30, 31]. Therefore, it will be of interest to test whether p21 downregulation resulting from the upregulation of CUL4 contributes to tumor activity. Many reports have shown that p21 accumulates during G1 phase but is degraded during S phase [32]. In addition, it is degraded after UV irradiation [33]. Nishitani et al. found the silence of CUL4, DDB1, CDT2 or PCNA by siRNA increased the level of p21 in S phase and inhibited its degradation following UV irradiation [34]. This suggests CUL4-DDB1^CDT2^ degrades p21 in PCNA-dependent way. In Cul4a conditional knockout mice, p21 protein increases [17], while the turnover of p21 is stabilized in DDB1^−/−^ cells [35]. These results provide proof for the accumulation of p21 observed in Cul4a^−/−^ cells. Silencing of CUL4B expression also increases p21 level in extra-embryonic cells [36] but has little effect in MEF cells [17]. Furthermore, Hall et al. found that CRL4^Cdt2^-mediated degradation of p21 was regulated by C/EBPα, the bZIP transcription factor, in response to UV-induced DNA damage. Following UV exposure, Cdt2 level failed to be down-regulated in C/EBPα-deficient keratinocytes, which consequently affected CRL4 to degrade p21 [37].

The turnover of p27 is tightly associated with cell cycle progression. Low expression of p27 would lead to rapid cell cycle progression and eventually induce oncogenesis. Multiple cancers have low level of p27 such as colon, prostate and ovarian tumor and so on. Moreover, downregulation of p27 relates to tumor patients prognosis [38]. The expression level of p27 could be modulated by CRL4 ^CDT2^ for degradation, and inactivation of CUL4A by siRNA is sufficient to cause the accumulation of p27, which results in G1 cell cycle arrest in treated cells [28, 39, 40]. Although many studies point that the level of p27 protein is controlled by CRL4, whether p27 is one direct substrate of CRL4 has been controversial. Consequently, further studies are needed to verify the above-mentioned question.

Additionally, Kotake et al. recently showed CUL4A could directly bind to p16, which was required for the latter activation [41]. And previous studies have suggested that p16 encodes a specific inhibitor of the CDK4 and CDK6, frequently mutated or inactivated in a majority of human tumors [42, 43]. Knockdown of the components of CRL4^Cdt2^ inhibits p12, the subunit of DNA pol δ, degradation in response to DNA damage, and p12 levels are reduced during the S phase under the control of CRL4^Cdt2^ during normal cell cycle progression [44-46]. The reduced expression of POLD4 encoding p12 protein has been found in lung cancer, which induces genomic instability [47].

Cdt1 is an important regulator of replication licensing and its regulation is critical for DNA replication once per cell cycle [48]. High level of Cdt1 contributes to aberrant replication and thus leads to malignant transformation [49]. So far, upregulation of Cdt1 has been found in many cancers such as colon and non-small-cell lung cancer. And Cdt1 overexpression could play a role in cancer development because its high level can occur early in premalignant diseases and participates in tumor development [50-52]. Cdt1 is degraded in response to different types of DNA lesions to ensure genomic stability. Cdt1 is present in G1 phase but at low levels after S phase by ubiquitin-mediated proteolysis. The work of Zhong et al. first provided proof that CRL4 complex degraded replication licensing factor Cdt1 during S phase to prevent re-replication [53]. Aberrant expression of CUL4 can be linked to unstable turnover of Cdt1 leading to DNA replication defects, aneuploidy and genomic instability and even cancer. In addition to CRL4, Cdt1 is also negatively regulated by geminin [54], SCF^Skp2^ complex [55] and APC [56]. The degradation of Cdt1 by CRL4 uses Cdt2 as substrate receptor, while the turnover of Cdt2 is also through ubiquitylation and then degradation. Studies found CRL1^FBXO11^ promoted the degradation of Cdt2 during S and G_2_/M phases.[57, 58] This suggests cross-talk between CRL1 and CRL4 ligase. CRL1^FBXO11^ degrades Cdt2 leading to the restraint of CRL4 activity, for example the degradation of p21 and Set8. Another factors related to re-replication also include p21 and PR-Set7/Set8 histone H4K20 methyltransferase that accumulates during G2 and M phase. The both substrates of CRL4 complex are degraded in S phase to prevent re-replication [59].

## CRL4 AND NER

Internal and external DNA-damaging agents induce DNA lesions, which constantly challenges DNA integrity. DNA lesions may lead to malignant transformation when such lesions are not repaired properly. Different DNA repair mechanisms remove different lesions and collectively safeguard genome stability. Nucleotide excision repair (NER) is a crucial pathway to detect and eliminate various lesions which mainly include two major lesions-cyclobutane pyrimidine dimers (CPD) and 6-4 pyrimidine-pyrimidone photoproducts (6-4PP) which are induced by UV irradiation [60]. NER includes two distinct subpathways: global genome NER (GG-NER), which removes lesions in the whole genome DNA, and transcription-coupled NER (TC-NER), which repairs DNA lesions in the transcribed strand of active genes [61]. A number of reports have shown that impaired NER can cause many distinct disorders including xeroderma pigmentosum, neurodegeneration and developmental disorders such as Cockayne syndrome and trichothiodystrophy [62]. Among the above disorders, XP patients are sun-sensitive and generally show a greatly increased incidence of UV-induced skin cancers. Two major CRL4 E3 ligase substrate receptors in NER are the Cockayne-syndrome protein A (CSA) and the damage DNA binding protein 2 (DDB2) involved in TCR and GGR respectively [63]. NER factors involved in GGR mainly include XPA-RPA, XPC-HR23B, and damage-specific DNA-binding proteins consisting of DDB1 and DDB2 subunits. Following UV, DDB1-DDB2 complex immediately localizes to the site of DNA damage and then recruits XPC to DNA damage sites. XPC is then ubiquitinated by CRL4^DDB2^ complex and subsequently deubiquitinated [64]. In Cul4a null MEFs, the levels of both DDB2 and XPC accumulate and dramatically enhance GGR activity. Furthermore, skin-specific Cul4a knockout mice increase resistance to UV-induced skin carcinogenesis because of elevated GGR [17]. Thus, inhibition of CRL4A ligase may provide a promising approach for UV-induced tumorigenesis. CSA and CSB are TCR factors, the mutation of which causes the Cockayne syndrome. Henning et al. reported that there was a physical interaction between CSA and CSB [65]. And CSA associates with CUL4A, which is involved in CRL4 E3 ligase [66]. Thus, CSB may be a substrate for CSA. In 2006, Groisman et al. showed that CUL4A^CSA^ E3 ligase activity up-regulation was accompanying with CSB ubiquitination and succeeding degradation after UV irradiation [67].

## CRL4 AND HISTONE METHYLATION

Tumor development not only depends on the genetic factors, but also associates with epigenetic modification [68]. Now epigenetic modification mainly refers to the studies involving in DNA methylation and histone modifications. Abnormal epigenetic alterations are involved in cancer development and progression. Recently, histone modifications have been reported in various cancers, and some histone alterations are found to be associated with poor prognosis [69, 70]. Histone modifications have abundant diversities which play important role in regulating a wide range of cellular processes such as transcription and genomic stability. Among them, histone methylation attaches more and more people's attention as a result of a host of evidence showing that there exists close relationship between dysfunction of histone methylation and tumor development [71]. Thus, the turnovers of factors associated with histone methylation would play important role in cancer. Higa et al. discovered CRL4 E3 ubiquitin ligase had critical effects on the regulation of histone methylation. They found that knockdown of CUL4 in HeLa cells resulted in reduced total level of H3K4me3, H3K9me3 and H3K27me3 [72]. And a recent study illustrated that CUL4A promoted H3K4me3 at the promoter of ZEB1 and then led to transcriptional upregulation of ZEB1 expression in breast cancer cells, and knockdown of ZEB1 blocked CUL4A-induced cell proliferation, EMT, tumorigenesis, and metastasis [14]. Thus, CUL4A can contribute to tumor development by the way of participating in histone methylation. Apart from CUL4A, latest work showed that CUL4B associated with Polycomb Repressive Complex 2 (PRC2) to play transcription repressive function through promoting H2AK119 monoubiquitination and H3K27 trimethylation at the promoter domain. The main target genes of CRL4B/PRC2 complex are p16 and PTEN, and CUL4B silencing results in increased expression of p16 and PTEN at both the transcriptional level and the protein level in KYSE410, HeLa, HEK293, MCF-7 (human breast adenocarcinoma cell line), and U2OS (human osteosarcoma cell line). Moreover, CUL4B/PRC2 complex promotes cell proliferation and invasion because of downregulating its target genes [21].

Additionally, recent researches also found CRL4 could degrade enzymes involved in histone methylation to affect histone modification. The loss of the histone methyltransferase PR-Set7/Set activity results in defective DNA replication and genomic instability during S phase [73]. This suggests that PR-Set7-mediated lysine methylation is a safeguarding factor for genome. PR-Set7 regulates the levels of H4K20 methylation, particularly for H4K20me1 and H4K20me3 [73]. And previous studies have shown that H4-K20me1 is associated with chromatin condensation [74] and loss of H4-K20me3 is observed in many human cancers [75]. In addition, Li, et al. found that SET8 participated in the Wnt signaling pathway [76], negatively regulated the function of p53 [77] and associated with TWIST activity, a master regulator of EMT [78], which suggested an interesting link between SET8 and carcinogenesis. Centore et al. suggested that Set8 was targeted by CRL4^Cdt2^ E3 ubiquitin ligase in a PCNA-dependent way for proteolysis in S phase and in response to DNA damage [59]. Furthermore at the same time, Abbas et al.[79] and Oda et al.[80] described similar points. Differences of the three groups are the exact time when PR-Set7 is degraded. Oda et al. found PR-Set7 is degraded during early G1 [80], while Abbas et al. and Centore et al. observed PR-Set7 is degraded specifically in S phase [59, 79]. A previous study also reported SCF/Skp2 is an E3 ligase for PR-Set7 [81]. Thus, future studies should deal with the cross-talk between CRL4/Cdt2 and SCF/Skp2 on the degradation of PR-Set7[82]. Given the regulation of PR-set7 on cell cycle, it will be interesting to explore whether PR-Set7 is a putative substrate for cellular transformation and contributes to tumor development. Furthermore, the regulation of CRL4 on the level of PR-set7 will provide proof to explore CRL4 in carcinogenesis.

## CRL4 AND TUMOR-RELATED SIGNALING PATHWAYS

As is known, carcinogenesis is related to abnormal signaling pathways. At present, many studies have found that CUL4 levels associate with aberrant signal transduction. For example, misregulation of the Wnt pathway has been found in many tumors, including colon cancer [83], gastric cancer [84], lung couancer [85] and so on. Abnormal regulation of components involved in Wnt pathway associates with cancer development. β-catenin, as one important part of Wnt signaling, is the key event in transduction of the Wnt signal, upregulation of which is the causes of many cancers [86]. When the Wnt signaling is activated, β-catenin accumulates and then migrates into nucleus to form a complex with the Lef-1/Tcf-1 in order to modulate the expression of target genes including some oncogenes such as c-myc [87, 88]. In the absence of Wnt signals, β-catenin is targeted to degradation by ubiquitin-proteasome pathway. In 1999, one paper showed that SCF complex used β-Trcp as substrate receptor to interact with β-catenin for its degradation. And serine phosphorylation of β-catenin is a prerequisite for its binding to β-Trcp [89]. Due to aberrant activation of the Wnt signaling pathway in tumor cells, β-catenin is no longer phosphorylated because of mutations in β-catenin itself or upstream elements that are critical for its phosphorylation, and thus escapes degradation. In 2007, another research found that CRL4B ligases could negatively regulate the expression of β-catenin [90]. Thus, the result supports the influence of CUL4B in Wnt regulation. However, so far there is no evidence to address the interplay between SCF/β-Trcp and CUL4B on the degradation of β-catenin. Interestingly, Yuan et al. recently found CUL4B played a positively regulatory role in Wnt/β-catenin signaling in hepatocellular carcinoma. They observed that in HCC tissues both CUL4B and β-catenin are overexpressed and the two are parallelly upregulated. *In vitro*, in CUL4B knockdown HCC cells, the level of total β-catenin protein was significantly reduced. With further experiments, they determined that CUL4B protected β-catenin from GSK3-mediated degradation in HCC cells, leading to the up-regulation of β-catenin [91]. In addition, the status of Wnt signaling pathway is also associated with some inhibitors. These inhibitors can prevent Wnt proteins from binding to their receptors or recruit some destruction complex to target β-catenin. The study found that CRL4B/PRC2 complexes promoted H2AK119me1 and H3K27me3 to repress expression of Wnt inhibitors, and thus activate Wnt signaling [91].

The mammalian target of rapamycin (mTOR), as a master regulator of cellular metabolism, proliferation, and survival, is a serine/threonine kinase forming two different and functionally distinct multiprotein complexes, mTORC1 and mTORC2 [92]. In many types of cancers, mTOR is aberrantly activated because of dysregulation of mTOR regulators or aberrant upstream signaling such as PI3K/AKT activation and PTEN loss. Recently, accumulating evidence suggests that CRL4 participates in the regulation of mTOR pathway by means of degradation or transcriptional repression of substrates related to mTOR. Merlin, a tumor suppressor and the protein product of the NF2 gene, plays important role in inhibition of cell proliferation and progression through the G1 phase of the cell cycle [93, 94]. Loss of merlin can induce the development of multiple nervous system tumors including schwannomas and meningiomas [95]. In 2009, one research found that merlin was a novel negative regulator of mTORC1 and merlin deficiency resulted in the activation of mTORC1 signaling in human meningioma cells [96]. And the work of Li et al. proved that merlin could inhibit the activity of CUL4A ubiquitin ligase by recruiting associated substrate receptor VprBP/DCAF1 [97, 98]. But other report held opposite opinion that the CRL4^VprBP^ ubiquitin ligase targeted merlin for degradation [99]. Thus the issue of the functional relationship between merlin and CRL4^VprBP^ is an important topic for future studies. In addition to merlin, CRL4 can also upregulate the mTOR pathway through promoting ubiquitination and degradation of tuberous sclerosis 2 (TSC2), a tumor suppressor, that forms a complex with TSC1 which can inhibit the mTOR signaling in a RheB-dependent manner [100, 101]. Moreover, researches indicated that TSC2 protein was identified for destruction by CRL4 E3 ubiquitin ligase *via* FBW5, a DDB1-binding WD40 protein. And overexpression of FBW5 or CUL4A promotes TSC2 protein degradation, while depletion of any department of FBW5, DDB1, or CUL4A/B stabilizes the turnover of TSC2 [101]. Lately, studies also found CUL4B repressed the expression of PTEN, the upregulator of mTOR [21].

Epidermal growth factor receptor (EGFR) has been found aberrant expression in many solid cancers [102-104]. Although ubiquitination pathway is the major mechanism involving in regulation of protein, the relationship between EGFR and CUL4 remains unclear. Lately, one study found in NSCLC cells CUL4A overexpression dramatically increased the level of EGFR transcript, while CUL4A silence markedly decreased the level of EGFR transcript [13]. Furthermore, CUL4A activates EGFR expression by the way of promoting H3K4 trimethylation. And they also found AKT, downstream target protein of EGFR, was increasingly phosphorylated due to CUL4A overexpression [13]. Thus, CUL4A may activated EGFR-AKT pathway, leading to NSCLC cells proliferation.

In addition to the above signal pathways, there are other signalings affected by CRL4 ligases. For example, CUL4B E3 ubiquitin ligase can degrade CSN5, while the latter has a crucial role in the regulation of BMP signaling by promoting the degradation of BMP inhibitor SMAD7 [105]. CRL4 can also regulate GRK5(G protein-coupled receptor kinase5) which binds with IκB inhibiting the NF-κB-mediated transcription [106]. CUL4A overexpresses and collaborates with H-Ras in the transformation of human mammary epithelial cells, which is consistent with the high frequency of RAS pathway activation in basal-like breast tumors [16].

## CRL4 AND ONCOPROTEINS

The reason of why CRL4 ligase is concerned with cancer development and progression is its substrates including a variety of oncoproteins. For example, the C-myc and N-myc proto-oncogenes belong to the family of myc genes that include B-myc, L-myc, and s-myc. The overexpression of the both myc genes is frequently found in various human cancers, which include breast carcinoma [107], lung carcinoma [108], and rare cases of colon carcinoma [109]. A recent report found a novel pathway that targeted myc proteins for degradation and was suppressed in cancer cells. That is the CRL4 E3 ligase complex recruits substrate receptor TRCP4AP/TRUSS for myc degradation through the proteasome and TRUSS knockdown leads to an increased level of myc proteins [110]. So TRCP4AP/TRUSS plays a key role as a myc-specific receptor for the CRL4 ligase complex, controlling the turnover of myc protein. In addition, CRL4 ligase degrades other oncoproteins through specific substrate receptors. For example, CRL4 respectively recruits Fbxw5 [111] and COP1 [112] as substrate receptor to target c-Myb and c-Jun for ubiquitination.

## CRL4 AND CANCER THERAPY

Because of the important role the ubiquitin system plays in cellular processes involved in cancer, development of drugs that modulate the activity of the system proves to be essential. Currently, bortezomib is the first and the only proteasome inhibitor approved by the US Food and Drug Administration for the treatment of multiple myeloma and cell lymphoma [113, 114]. However, bortezomib generally inhibits proteasome function, in return there are many side effects. Thus, it is more valuable to develop a relatively specific means to modulate levels of important proteins. In ubiquitin-proteasome process, E3 ligases determine the substrate specificity, so targeting of specific E3s has the potential to selectively stabilize specific cellular proteins and would theoretically avoid side effects. MLN4924, a newly discovered small molecule inhibitor of NEDD8-activating enzyme, inactivates CRL E3 ligases and consequently causes accumulation of CRLs substrates and suppresses tumor cell growth both *in vitro* and *in vivo* [115]. Thanks to selectively blocking degradation of a specific set of proteins regulated by CRL E3s, MLN4924-induced cytotoxicity is less than that of targeting proteasomes by bortezomib. However, MLN4924 affects a broad range of substrates targeted by multiple CRLs E3 for degradation, which is similar to proteasome inhibition. Thus, the specific inhibition of individual CRLs may be more effective and provides a better therapeutic index than global inhibition *via* MLN4924.

Recent studies discussed above have shown that CRL4 plays essential roles in cancer. Overexpression of CUL4A/B has been demonstrated in many types of cancers [9-12, 21, 22], and is associated with a poor prognosis of patient survival [15, 20]. Moreover, knockdown of CUL4A/B inhibits the growth of cancer cells [16, 91, 116, 117], while CUL4A/B overexpression promotes malignant proliferation [14, 21, 118, 119]. Given these facts, CRL4 may be a potential target for cancer therapy.

Colon cancer is a common gastrointestinal cancer, and chemotherapy is a major treatment program for advanced cases. Irinotecan is one specific topoisomerase I (TOP1) inhibitor, but down-regulation of TOP1 has been found in some colon cancer cases, leading to reduced therapeutic effect of irinotecan. Researches found mutation of CUL4B decreased TOP1 ubiquitination and cells defective CUL4B exhibited sensitivity to camptothecin [120]. Thus, specific inhibitors of CUL4B could increase the level of TOP1 and consequently improve therapeutic effect of irinotecan on colon cancer. Pan et al. found ovarian cancer cells with overexpression of CRL4 were more sensitive to MLN4924 treatment than normal cells, such as ovarian surface epithelial cells and knockdown of CRL4 components in ovarian cancer cells mimicked the growth inhibition effects of MLN4924 [121]. Consequently, overexpression of CRL4 components is expected to be a useful biomarker for ovarian cancer and determines MLN4924 efficacy in cancer treatment. In addition, prostate cancer cells with high expression of CUL4A are sensitive to thalidomide, and apoptosis to thalidomide also depends on high level of CUL4A [122]. Screening of CUL4A level in prostate cancer patients benefits to determine whether some patients should accept therapy of thalidomide. Cereblon (CRBN) was first identified by Higgins et al. in patients with autosomal recessive nonsyndromic mental retardation [123]. Recently, studies found the level of CRBN in myeloma cells was associated with better treatment response in IMiDs (thalidomide, lenalidomide and pomalidomide) treatment [124, 125]. Further studies found CRBN interacted with DDB1, CUL4 and ROC1 to form CRL4^Cereblon^ E3 complex [126]. IMiDs directly bind Cereblon (CRBN), and promote substrates Ikaros and Aiolos to the CRL4^Cereblon^ E3 complex for degradation, which is toxic to myeloma cells [127]. Overexpression of CDT2, one substrate receptor of CRL4, has been reported in many types of tumors such as breast [128], gastric [129], and ovarian carcinomas [121]. Silencing of CDT2 results in apoptotic death in cancer cells but not in non-transformed cells [130]. This suggests loss of CDT2 affects viability of cancer cells. Previous studies have found CRL4^CDT2^ targets many substrates for destruction associated with cancer that include CDT1 [131], p21 [34] and CHK1 [132]. Consequently, down-modulation of CDT2 may be a promising approach to cancer therapy.

In addition, many works (discussed above) have found CRL4 participates in the regulations of signaling pathways associated with cancer. Specific inhibitors of CRL4 will change the status of these pathways, leading to get tumor inhibitory effects. For example, mTOR pathway has been found activated in a wide variety of diseases including cancer [92]. Thus, mTOR inhibitors such as temsirolimus [133] and Torin1 [134] may have a broad application in the treatment of cancer. Apart from this, modulating the turnover of mTOR-negative regulators may be an alternative approach of blocking mTOR pathway. Previous studies found TSC2 and REDD1, two mTOR-negative regulators, are degraded by CRL4^FBW5^ ligase and CRL4^β-Trcp^ ligase respectively [101, 135]. Small-molecule inhibitors of the CRL4 E3s are expected to cause their accumulation, leading to the inhibition of mTOR pathway.

Specific inhibitors of SCF E3s have been proved as the attractive anticancer targets [136]. Given both CRL4 E3s and SCF E3s belonging to the same family, so it is promising that specific inhibitors of CRL4 E3s as novel anticancer agents will be eventually discovered.

## CONCLUSIONS

A significant number of recent works shed light on the biological functions of CRL4 E3 ligases that play a vital role in tumor development and progression. CRL4 ligases have been shown to degrade diverse substrates associated with a wide range of cellular processes that influence the course of cancer development and progression, including the cell cycle, genomic stability, transcriptional suppression, histone methylation and so on. Hence, given the roles of CRL4 ligases in tumorigenesis, CRL4-mediated ubiquitination pathways possess broad prospects in intervention and prevention of carcinogenesis. Nevertheless, even so many studies of cancer-relevant CRL4 ligases, there are still many controversies to be answered. For instance, more studies are apparently needed to further distinguish the functional distinction between CUL4A and CUL4B in cancer. Further works are essential to resolve the puzzling role of CRL4 ligases in the maintenance of genome stability. More molecules related to cancer need to be proved whether they are the substrates of CUL4. Additionally, drug specificity is also one urgent problem to resolve. Although many challenges are needed to represent the role of CRL4 ligases in tumor, a considerable wealth of information has been generated regarding the mechanisms of CRL4 ligases' biological function and their clinical implications in cancer. Moreover, more and more studies show that it will be a promising novel target for cancer therapy. Consequently, works on CRL4 ligases in cancer deserve more concerns in the future.
